# Supplementary material for: The relationship between maternal uterine artery doppler ultrasound during the second and third trimesters and infant cognitive development at one year of age: Findings from a South African prospective cohort study
Source: PLoS One. 2025 Jun 6;20(6):e0322806. doi: 10.1371/journal.pone.0322806 (PMC12143890; doi:10.1371/journal.pone.0322806)
Supplement: Attachment — Submitted filename: Response to Reviewers.docx [file pone.0322806.s004.docx]

**Reviewers’ comments**

We would like to thank the editors and reviewers for their helpful comments. Each point raised have been addressed below and as track changes on the manuscript as well.

**Journal Requirements:**

**1. When submitting your revision, we need you to address these additional requirements.

Please ensure that your manuscript meets PLOS ONE's style requirements, including those for file naming. The PLOS ONE style templates can be found at**
[**https://journals.plos.org/plosone/s/file?id=wjVg/PLOSOne_formatting_sample_main_body.pdf**](https://eur02.safelinks.protection.outlook.com/?url=https%3A%2F%2Fjournals.plos.org%2Fplosone%2Fs%2Ffile%3Fid%3DwjVg%2FPLOSOne_formatting_sample_main_body.pdf&data=05%7C02%7C%7C0226d3bc9c274dbb349c08dd5060b545%7C2e9f06b016694589878910a06934dc61%7C0%7C0%7C638755098934717071%7CUnknown%7CTWFpbGZsb3d8eyJFbXB0eU1hcGkiOnRydWUsIlYiOiIwLjAuMDAwMCIsIlAiOiJXaW4zMiIsIkFOIjoiTWFpbCIsIldUIjoyfQ%3D%3D%7C0%7C%7C%7C&sdata=TGyTZGX2Kr7%2BACMd7L%2F2LHb%2B3MHF%2FmHn2JaZkcro1x0%3D&reserved=0) **and**
[**https://journals.plos.org/plosone/s/file?id=ba62/PLOSOne_formatting_sample_title_authors_affiliations.pdf**](https://eur02.safelinks.protection.outlook.com/?url=https%3A%2F%2Fjournals.plos.org%2Fplosone%2Fs%2Ffile%3Fid%3Dba62%2FPLOSOne_formatting_sample_title_authors_affiliations.pdf&data=05%7C02%7C%7C0226d3bc9c274dbb349c08dd5060b545%7C2e9f06b016694589878910a06934dc61%7C0%7C0%7C638755098934733462%7CUnknown%7CTWFpbGZsb3d8eyJFbXB0eU1hcGkiOnRydWUsIlYiOiIwLjAuMDAwMCIsIlAiOiJXaW4zMiIsIkFOIjoiTWFpbCIsIldUIjoyfQ%3D%3D%7C0%7C%7C%7C&sdata=PvXCXJw5%2B%2F869ianHaex5%2Fz%2F73khQTNKfcpN%2BTojIKQ%3D&reserved=0)

We have carefully reformatted the manuscript to align with **PLOS ONE’s** style requirements, ensuring compliance throughout. To the best of our knowledge, the only remaining formatting consideration is the reference style. We have formatted the references using **PLOS ONE’s** citation style in **EndNote**, which displays in-text reference numbers as superscripts. We hope this is acceptable, but we are happy to make any further adjustments as needed.

Thank you for your guidance. **2. Please include a complete copy of PLOS’ questionnaire on inclusivity in global research in your revised manuscript. Our policy for research in this area aims to improve transparency in the reporting of research performed outside of researchers’ own country or community. The policy applies to researchers who have travelled to a different country to conduct research, research with Indigenous populations or their lands, and research on cultural artefacts. The questionnaire can also be requested at the journal’s discretion for any other submissions, even if these conditions are not met. Please find more information on the policy and a link to download a blank copy of the questionnaire here:** [**https://journals.plos.org/plosone/s/best-practices-in-research-reporting**](https://eur02.safelinks.protection.outlook.com/?url=https%3A%2F%2Fjournals.plos.org%2Fplosone%2Fs%2Fbest-practices-in-research-reporting&data=05%7C02%7C%7C0226d3bc9c274dbb349c08dd5060b545%7C2e9f06b016694589878910a06934dc61%7C0%7C0%7C638755098934749693%7CUnknown%7CTWFpbGZsb3d8eyJFbXB0eU1hcGkiOnRydWUsIlYiOiIwLjAuMDAwMCIsIlAiOiJXaW4zMiIsIkFOIjoiTWFpbCIsIldUIjoyfQ%3D%3D%7C0%7C%7C%7C&sdata=FDzhSxFvi1F32mUKUSgWfFWm45yPoL8Q6CSzyYeL23I%3D&reserved=0)**. Please upload a completed version of your questionnaire as Supporting Information when you resubmit your manuscript.**

We have completed the **PLOS ONE** questionnaire on inclusivity in global research to the best of our ability and where relevant. The completed form has been attached as **supporting information** in this revised submission. **3. Please include your tables as part of your main manuscript and remove the individual files. Please note that supplementary tables (should remain/ be uploaded) as separate "supporting information" files.**

We have incorporated the two tables into the **main manuscript** as requested. Table 1 is located at line 140 and Table 2 is at line 269.  **4. In the online submission form, you indicated that [Data can be made available through request to the corresponding author].

All PLOS journals now require all data underlying the findings described in their manuscript to be freely available to other researchers, either 1. In a public repository, 2. Within the manuscript itself, or 3. Uploaded as supplementary information.

This policy applies to all data except where public deposition would breach compliance with the protocol approved by your research ethics board. If your data cannot be made publicly available for ethical or legal reasons (e.g., public availability would compromise patient privacy), please explain your reasons on resubmission and your exemption request will be escalated for approval.**

The complete data set of the full study will be available on the National Institute of Child Health and Human Development Data and Specimen Hub Data (NICHD DASH) with free access to the public.

The data is not available in the manuscript or as supplementary material but could be requested from the authors. **5. Thank you for stating the following financial disclosure: [NIAAA and NIDCD (U01 AA016501)].

Please state what role the funders took in the study. If the funders had no role, please state: ""The funders had no role in study design, data collection and analysis, decision to publish, or preparation of the manuscript.""
If this statement is not correct you must amend it as needed.

Please include this amended Role of Funder statement in your cover letter; we will change the online submission form on your behalf.**

We have added the following statement to the funding section on the title page:

“The funders were involved in the design of the original SPS. The funders had no role in the study design, data collection and analysis, decision to publish or preparation of the manuscript.”

Thank you for your guidance. Please let us know if any further revisions are required.

**6. Thank you for stating the following in your Competing Interests section: [No].

Please complete your Competing Interests on the online submission form to state any Competing Interests. If you have no competing interests, please state ""The authors have declared that no competing interests exist."", as detailed online in our guide for authors at** [**http://journals.plos.org/plosone/s/submit-now**](https://eur02.safelinks.protection.outlook.com/?url=http%3A%2F%2Fjournals.plos.org%2Fplosone%2Fs%2Fsubmit-now&data=05%7C02%7C%7C0226d3bc9c274dbb349c08dd5060b545%7C2e9f06b016694589878910a06934dc61%7C0%7C0%7C638755098934765721%7CUnknown%7CTWFpbGZsb3d8eyJFbXB0eU1hcGkiOnRydWUsIlYiOiIwLjAuMDAwMCIsIlAiOiJXaW4zMiIsIkFOIjoiTWFpbCIsIldUIjoyfQ%3D%3D%7C0%7C%7C%7C&sdata=Ro27AP%2FpKCV36jyTEdFo6RtmhGu7C4vAHJ7lVykIX5Y%3D&reserved=0) **This information should be included in your cover letter; we will change the online submission form on your behalf.**

We have included a statement of no competing interests in the cover letter attached to this revised submission. Please let us know if further revisions are needed.  **7. Please include captions for your Supporting Information files at the end of your manuscript, and update any in-text citations to match accordingly. Please see our Supporting Information guidelines for more information:** [**http://journals.plos.org/plosone/s/supporting-information**](https://eur02.safelinks.protection.outlook.com/?url=http%3A%2F%2Fjournals.plos.org%2Fplosone%2Fs%2Fsupporting-information&data=05%7C02%7C%7C0226d3bc9c274dbb349c08dd5060b545%7C2e9f06b016694589878910a06934dc61%7C0%7C0%7C638755098934781828%7CUnknown%7CTWFpbGZsb3d8eyJFbXB0eU1hcGkiOnRydWUsIlYiOiIwLjAuMDAwMCIsIlAiOiJXaW4zMiIsIkFOIjoiTWFpbCIsIldUIjoyfQ%3D%3D%7C0%7C%7C%7C&sdata=Xkl%2Fbv0r8E9NaKQO5X8pd0pSoAflh%2BoFHjzM0KunBO4%3D&reserved=0)**.**

We have included captions for Supporting Information files at the end of the manuscript (line 421).

**8. Please review your reference list to ensure that it is complete and correct. If you have cited papers that have been retracted, please include the rationale for doing so in the manuscript text, or remove these references and replace them with relevant current references. Any changes to the reference list should be mentioned in the rebuttal letter that accompanies your revised manuscript. If you need to cite a retracted article, indicate the article’s retracted status in the References list and also include a citation and full reference for the retraction notice.**

The reference list has been reviewed and to the best of our knowledge is complete and correct. If there are any references that the editor believes should be removed, please do let us know.

**Additional Editor Comments:

The manuscript titled "The Relationship Between Maternal Uterine Artery Doppler Ultrasound During the Second and Third Trimesters and Infant Cognitive Development at One Year of Age: Findings from a South African Prospective Cohort Study" is well-written and clear. The study’s aim is compelling, and the methodology is appropriate. I enjoyed reading it.

To enhance the manuscript, I suggest the following revisions:

Please avoid using abbreviations in the abstract.**

We have revised the abstract to remove all abbreviations, ensuring clarity and alignment with journal guidelines.

**In the Ethics section, specify whether consent for participation and publication was obtained, given the prospective nature of the study.**

We have added the following statement to the Ethics section (lines 220-222):

*“Participants provided written informed consent, including approval for the use of their data in future studies. Data from those who did not provide consent were not used”.*

**Expanding on the clinical and research implications in the Discussion section would be beneficial.**

We appreciate this valuable suggestion and have expanded on the clinical implications in the Discussion section. Specifically, we have emphasized the importance of obstetricians and maternal-fetal specialists being aware of the potential for developmental delay in children with abnormal uterine Doppler findings during pregnancy. This awareness can help ensure timely assessments and early intervention where needed (Discussion, lines 390-93).

We also sincerely thank the editor for their time and consideration in reviewing our manuscript. **Reviewers' comments:

Reviewer's Responses to Questions**

**Comments to the Author

1. Is the manuscript technically sound, and do the data support the conclusions?

The manuscript must describe a technically sound piece of scientific research with data that supports the conclusions. Experiments must have been conducted rigorously, with appropriate controls, replication, and sample sizes. The conclusions must be drawn appropriately based on the data presented.**

**Reviewer #1: Yes**

**Reviewer #2: Yes**

**2. Has the statistical analysis been performed appropriately and rigorously?**

**Reviewer #1: Yes**

**Reviewer #2: Yes**

**3. Have the authors made all data underlying the findings in their manuscript fully available?

The** [**PLOS Data policy**](https://eur02.safelinks.protection.outlook.com/?url=http%3A%2F%2Ftrack.editorialmanager.com%2FCL0%2Fhttp%3A%252F%252Fwww.plosone.org%252Fstatic%252Fpolicies.action%2523sharing%2F1%2F010f01951a823315-33b8f17a-e469-4027-a449-ac1dd6cc96d8-000000%2FrOx700xxbO49z8Ao-dh2mIkstsVGlk-E6uMVPPbUQN8%3D198&data=05%7C02%7C%7C0226d3bc9c274dbb349c08dd5060b545%7C2e9f06b016694589878910a06934dc61%7C0%7C0%7C638755098934797787%7CUnknown%7CTWFpbGZsb3d8eyJFbXB0eU1hcGkiOnRydWUsIlYiOiIwLjAuMDAwMCIsIlAiOiJXaW4zMiIsIkFOIjoiTWFpbCIsIldUIjoyfQ%3D%3D%7C0%7C%7C%7C&sdata=9rbqAIG7ZDFlnDhbRch2VWo9c%2Ftkdwo9C1dXJGe%2FPU4%3D&reserved=0) **requires authors to make all data underlying the findings described in their manuscript fully available without restriction, with rare exception (please refer to the Data Availability Statement in the manuscript PDF file). The data should be provided as part of the manuscript or its supporting information, or deposited to a public repository. For example, in addition to summary statistics, the data points behind means, medians and variance measures should be available. If there are restrictions on publicly sharing data—e.g. participant privacy or use of data from a third party—those must be specified.**

**Reviewer #1: Yes**

**Reviewer #2: Yes**

**4. Is the manuscript presented in an intelligible fashion and written in standard English?

PLOS ONE does not copyedit accepted manuscripts, so the language in submitted articles must be clear, correct, and unambiguous. Any typographical or grammatical errors should be corrected at revision, so please note any specific errors here.**

**Reviewer #1: Yes**

**Reviewer #2: Yes**

**5. Review Comments to the Author

Please use the space provided to explain your answers to the questions above. You may also include additional comments for the author, including concerns about dual publication, research ethics, or publication ethics. (Please upload your review as an attachment if it exceeds 20,000 characters)**

**Reviewer #1: the manuscript is interesting despite not only UtA are correlated with neurodevelopmentall delay but also birth whicht, inasnuch the null hypothesis of the study has to be changed and highlight the outcomes better for reders**

We sincerely appreciate your thoughtful feedback and for recognizing the value of our manuscript. Regarding your comment on the null hypothesis, we would like to clarify that our study hypothesis is explicitly stated in the final paragraph of the introduction.

**Reviewer #2: The authors have done an excellent job in writing this article. It is well-written, and the subject matter is highly relevant. However, two revisions are recommended. First, a paragraph discussing the clinical implications of the findings should be included. Second, further elaboration on suggestions for future research would strengthen the manuscript, particularly expanding on the potential for studies with larger sample sizes, which is briefly mentioned.**

We thank Reviewer #2 for their helpful revisions, which we agree will improve the quality of our manuscript. We have added a comment on the clinical implications of the findings: “Obstetricians and Maternal-Fetal specialists should be informed of the possibility of developmental delay in children when abnormal uterine Doppler assessments had been recorded in the mother during the pregnancy, to ensure that these children are carefully assessed at appropriate times and remedial treatment instituted when necessary.” (line 378-81)

We have further amended the title of the ‘Strengths and Limitations’ section within the Discussion to be ‘Strengths, Limitations and Future Directions’ to accurately reflect the content of this sub-section. The following points have been added to this section to reflect on future directions for research, with a focus on sample size of future studies:

- “It would be interesting to conduct a study of similar design which stratifies participants by term delivery to examine how much of the effect observed is due to preterm birth.” (line 305-6)
- “Future research with larger sample sizes is required to elucidate the associations between placental function and offspring cognitive development. Larger sample sizes will afford the findings with increased statistical power and provide the opportunity to include a truly generalisable sample.” (line 308-11)

**6. PLOS authors have the option to publish the peer review history of their article (**[**what does this mean?**](https://eur02.safelinks.protection.outlook.com/?url=https%3A%2F%2Ftrack.editorialmanager.com%2FCL0%2Fhttps%3A%252F%252Fjournals.plos.org%252Fplosone%252Fs%252Feditorial-and-peer-review-process%2523loc-peer-review-history%2F1%2F010f01951a823315-33b8f17a-e469-4027-a449-ac1dd6cc96d8-000000%2FlcvEJ1mgggi79-jabo0WMNUPDqaKc-htyy4RvAPzO0c%3D198&data=05%7C02%7C%7C0226d3bc9c274dbb349c08dd5060b545%7C2e9f06b016694589878910a06934dc61%7C0%7C0%7C638755098934813931%7CUnknown%7CTWFpbGZsb3d8eyJFbXB0eU1hcGkiOnRydWUsIlYiOiIwLjAuMDAwMCIsIlAiOiJXaW4zMiIsIkFOIjoiTWFpbCIsIldUIjoyfQ%3D%3D%7C0%7C%7C%7C&sdata=%2Fd7anvh1jQibpeKiKO1SvOhd2VKGYwOIpWG5Cx4%2FwOo%3D&reserved=0)**). If published, this will include your full peer review and any attached files.


If you choose “no”, your identity will remain anonymous but your review may still be made public.


Do you want your identity to be public for this peer review? For information about this choice, including consent withdrawal, please see our** [**Privacy Policy**](https://eur02.safelinks.protection.outlook.com/?url=https%3A%2F%2Ftrack.editorialmanager.com%2FCL0%2Fhttps%3A%252F%252Fwww.plos.org%252Fprivacy-policy%2F1%2F010f01951a823315-33b8f17a-e469-4027-a449-ac1dd6cc96d8-000000%2F6xyw4MWfJDsxMo-xvbtxvriqiuM2rUQ4BVGI8JdMeQk%3D198&data=05%7C02%7C%7C0226d3bc9c274dbb349c08dd5060b545%7C2e9f06b016694589878910a06934dc61%7C0%7C0%7C638755098934830249%7CUnknown%7CTWFpbGZsb3d8eyJFbXB0eU1hcGkiOnRydWUsIlYiOiIwLjAuMDAwMCIsIlAiOiJXaW4zMiIsIkFOIjoiTWFpbCIsIldUIjoyfQ%3D%3D%7C0%7C%7C%7C&sdata=POvnW4lcHRuZybR6ZxnVPgU%2FISEpgVVDv%2BFyzsKjN9o%3D&reserved=0)**.**

**Reviewer #1: Yes: Erich Cosmi**

**Reviewer #2: No**

Thank you again for reviewing our manuscript and for the points raised.
